# Women and the Heart: Webcast April 16 2024

**DOI:** 10.14797/mdcvj.1525

**Published:** 2024-12-26

**Authors:** Miguel A. Quiñones, Valeria E. Duarte, Christopher Hobday, Saurabh Rajpal, Carla P. Rodriguez-Monserrate, Karla Kurrelmeyer, Gladys Velarde

**Affiliations:** 1 Houston Methodist DeBakey Heart & Vascular Center, Houston, Texas, US; 2 Boston Children’s Hospital, Harvard Medical School, Boston, Massachusetts, US; 3 Obstetrics and Gynecology Department, Houston Methodist, Houston, Texas, US; 4 The Ohio State University Wexner Medical Center, Columbus, Ohio, US; 5 Nationwide Children’s Hospital, Columbus, Ohio, US; 6 Boston Children’s Hospital and Brigham and Women’s Hospital, Harvard Medical School, Boston, Massachusetts, US; 7 Methodist DeBakey Cardiology Associates, Houston Methodist Hospital, Houston, Texas, US; 8 University of Florida College of Medicine, Jacksonville, Florida, US

**Keywords:** pregnancy, congenital heart disease, arrhythmia in pregnancy, thoracic aortic disease, sex-specific etiologies, cardiomyopathy, pulmonary hypertension, estrogen paradox, ischemic heart disease

## Abstract

This 62-minute webcast features a conversation about “Women and the Heart: Gender-Related Differences in Cardiovascular Care”—the focus of Issue 20.2. Led by the issue’s editor, the discussion engages the authors on emerging themes and lessons learned while researching and writing the articles. View the video at https://livestream.com/accounts/21157318/events/11132857.

## Introduction

This 62-minute webcast (Video 1) features the *Journal*’s editor-in-chief and the issue’s guest editor conversing with four of the issue’s authors on the emerging themes and lessons learned while exploring the article topics related to “Women and the Heart: Gender-Related Differences in Cardiovascular Care.” The following list outlines where to view guests and topics:

**Video 1:** The editors and authors discuss details and explore overarching themes in Issue 20.2, titled “Women and The Heart.”

### Welcome

Miguel Quiñones, MD, introduces topic (2:15)

Dr. Quiñones introduces guest editor Valeria Duarte, MD (3:50)

Dr. Duarte offers in-depth overview of topics (4:38)

**DR. DUARTE INTRODUCES AUTHORS**

Christopher Hobday, MD (6:57)

Karla Kurrelmeyer, MD (7:24)

Gladys Velarde, MD (7:513)

Saurabh Rajpal, MD (8:22)

Carla P. Rodriguez-Monserrate, MD, FACC (8:27)

**“HYPERTENSIVE DISORDERS OF PREGNANCY”**

Dr. Quiñones begins (8:50)

Dr. Christopher Hobday begins (9:52)

**“HIGH-RISK CONGENITAL HEART DISEASE IN PREGNANCY”**

Dr. Duarte begins (15:20)

Dr. Rajpal begins (16:18)

Dr. Rodriguez begins (25:53)

Dr. Quinones asks about pregnancy with ACHD and co-morbidities (29.42)

**“SPECTRUM OF ISCHEMIC HEART DISEASE THROUGHOUT A WOMAN’S LIFE CYCLE”**

Dr. Duarte begins (35:00)

Dr. Kurrelmeyer begins (35:58)

**“THE IMPACT OF PREGNANCY IN PATIENTS WITH THORACIC AORTIC DISEASE: EPIDEMIOLOGY, RISK ASSESSMENT, AND MANAGEMENT CONSIDERATIONS”**

Dr. Quiñones begins (42:55)

Dr. Duarte begins (43:39)

**“SEX DISPARITIES IN CARDIOVASCULAR DISEASE”**

Dr. Duarte begins (48:42)

Dr. Velarde begins (49:45)

## Conclusion

Dr. Quiñones wrap-up (58:10)

Dr. Duarte wrap-up (58:33)

